# Amide proton transfer MRI differentiates between progressive multifocal leukoencephalopathy and malignant brain tumors: a pilot study

**DOI:** 10.1186/s12880-022-00959-3

**Published:** 2022-12-26

**Authors:** Hirofumi Koike, Minoru Morikawa, Hideki Ishimaru, Reiko Ideguchi, Masataka Uetani, Takeshi Hiu, Takayuki Matsuo, Mitsuharu Miyoshi

**Affiliations:** 1 Department of Radiology, Nagasaki University Graduate School of Biomedical Sciences, 1-7-1 Sakamoto, Nagasaki, 852-8501 Japan; 2 Department of Radiology, Nagasaki University Hospital, 1-7-1 Sakamoto, Nagasaki, 852-8501 Japan; 3 Department of Radioisotope Medicine, Nagasaki University Graduate School of Biomedical Sciences, 1-7-1 Sakamoto, Nagasaki, 852-8588 Japan; 4 Department of Neurosurgery, Nagasaki University Graduate School of Biomedical Sciences, 1-7-1 Sakamoto, Nagasaki, 852-8501 Japan; 5 MR Application and Workflow, GE Healthcare Japan, 4-7-127 Asahigaoka, Hino, Tokyo 191-8503 Japan

**Keywords:** Magnetic resonance imaging, Amide proton transfer imaging, Progressive multifocal leukoencephalopathy, Primary central nervous system lymphoma, Glioblastoma multiforme

## Abstract

**Background:**

Progressive multifocal leukoencephalopathy (PML) is a demyelinating disease of the central nerve system caused by the John Cunningham virus. On MRI, PML may sometimes appear similar to primary central nervous system lymphoma (PCNSL) and glioblastoma multiforme (GBM). The purpose of this pilot study was to evaluate the potential of amide proton transfer (APT) imaging for differentiating PML from PCNSL and GBM.

**Methods:**

Patients with PML (n = 4; two men; mean age 52.3 ± 6.1 years), PCNSL (n = 7; four women; mean age 74.4 ± 5.8 years), or GBM (n = 11; 6 men; mean age 65.0 ± 15.2 years) who underwent APT-CEST MRI between January 2021 and September 2022 were retrospectively evaluated. Magnetization transfer ratio asymmetry (MTR_asym_) values were measured on APT imaging using a region of interest within the lesion. Receiver operating characteristics curve analysis was used to determine diagnostic cutoffs for MTR_asym_.

**Results:**

The mean MTR_asym_ values were 0.005 ± 0.005 in the PML group, 0.025 ± 0.005 in the PCNSL group, and 0.025 ± 0.009 in the GBM group. There were significant differences in MTR_asym_ between PML and PCNSL (*P* = 0.023), and between PML and GBM (*P* = 0.015). For differentiating PML from PCNSL, an MTR_asym_ threshold of 0.0165 gave diagnostic sensitivity, specificity, positive predictive value, and negative predictive value of 100% (all). For differentiating PML from GBM, an MTR_asym_ threshold of 0.015 gave diagnostic sensitivity, specificity, positive predictive value, and negative predictive value of 100%, 90.9%, 80.0%, and 100%, respectively.

**Conclusion:**

MTR_asym_ values obtained from APT imaging allowed patients with PML to be clearly discriminated from patients with PCNSL or GBM.

## Background

Progressive multifocal leukoencephalopathy (PML) is a demyelinating disease of the brain resulting from lytic infection of glial cells due to the ubiquitous human polyomavirus 2, the John Cunningham virus (JCV), which is present in a quiescent state in up to 80% of the adult population [1]. In conditions of immunocompromise, such as in acquired immunodeficiency syndrome (AIDS), hematological malignancies, or immunosuppressive treatments, reactivation of JCV may occur, leading to PML [2]. Some cases of PML have also been reported in patients with only mild immunosuppression, such as in patients with hepatic cirrhosis [3] and in patients with no immunosuppression [4]. Characteristic magnetic resonance imaging (MRI) features of PML include hyperintense lesions on T2-weighted imaging (T2WI) and fluid attenuated inversion recovery (FLAIR) imaging, which are asymmetric, show an absence of contrast enhancement or mass effect, and relatively spare periventricular white matter [5]. Primary central nervous system lymphoma (PCNSL) sometimes looks like PML on MRI, with abnormalities confined to the white matter and devoid of a mass effect. A few cases of PCNSL showed non enhancing mass lesions [6–8]

Moreover, PCNSL, like PML, occurs with immunosuppression [9], and an association between these two diseases has been described [10]. Glioblastoma multiforme (GBM) is the most common and aggressive primary brain tumor in adults, accounting for approximately 15% of all intra-cranial tumors [11], and PML can rarely mimic multifocal glioma progression on MRI [12]. There was also a report of PML that is pathologically accompanied by bleeding and has a contrast enhancement effect on MRI [13]

Amide proton transfer (APT) imaging is a novel chemical exchange saturation transfer (CEST) MRI technique [14, 15] deriving contrast from the amide protons of endogenous low-concentration mobile proteins and peptides in tissues, such as those dissolved in the cytoplasm [16, 17]. APT can be evaluated quantitatively, and in neuroimaging it has shown potential clinical utility for analysis of brain tumors [18–23], cerebral ischemia [24–26], and Parkinson’s disease [27]. APT imaging does not require exogenous contrast and is safe in patients with renal failure or with intolerance to contrast media. Although the origin of the APT signal intensity (SI) in lesions has not been fully explained, it has been attributed to increased mobile protein concentrations in cells [18, 20, 21, 23, 28, 29]. However, there are few reports on the application of APT SI in demyelinating disease, even though it is important to distinguish between demyelinating disease (including PML) and malignant tumors because of the different treatment required.

Therefore, the purpose of the present study was to investigate differences in the APT signals of PML in comparison with those of PCNSL and GBM. We also aimed to determine the value of APT imaging for differentiating PML from PCNSL and GBM.

## Methods

### Patients

This retrospective study recruited 22 patients (11 men [50%] and 11 women [50%]; mean age [standard deviation {SD}]: 65.7 years [13.8]) who underwent MRI with APT imaging in our hospital because of suspected brain tumor between January 2021 and September 2022. At our institution, APT imaging is now part of the initial MRI acquisition for patients with suspected brain tumor.

Our institutional review board approved this study and waived the need for written informed consent because of the retrospective design. However, information describing that all patient data would be used for research purposes was posted on the hospital’s homepage, giving patients the opportunity to reject the use of their data.

### MRI protocol

MRI was performed on a 3-T clinical scanner (Signa™ Architect, GE Healthcare, Milwaukee, WI, USA) with a 48-channel receiver array coil. The MRI protocols used in this study were vendor-supplied protocols. Axial two-dimensional (2D) APT imaging was acquired before contrast administration using a single-shot fast spin echo (SSFSE) pulse sequence with radiofrequency (RF) saturation (one pulse with a duration of 2000 ms, and average B_1_ radiofrequency field equivalent to a continuous RF power level of 2.0 µT). The imaging parameters used were: field of view = 220 × 220 mm, matrix = 128 × 128, spatial resolution = 1.7 × 1.7 mm, slice thickness = 8.0 mm, repetition time/echo time (TR/TE) = 3000/26.6 ms, and number of slices = 1. Twenty-nine saturation frequency offsets from − 7.0 to 7.0 ppm in increments of 0.5 ppm were adopted to attain a sufficient signal-to-noise ratio within the clinical time frame. Water-frequency shift owing to field inhomogeneity was measured in a separate image acquired using the water-saturation shift referencing method with 11 offset frequencies ranging from − 1.875 to 1.875 ppm at intervals of 0.375 ppm, with one reference image acquired without a saturation RF pulse, resulting in a full Z-spectrum within the offset range. The water-saturation shift reference image was obtained with a TR/TE of 3000/26.6 ms, RF saturation amplitude of 0.5 µT, and total duration of 2000 ms, with a continuous wave. The total acquisition time for both APT and water-saturation shift reference images was 2 min 9 s.

Other conventional MRI sequences were acquired following the standard brain tumor protocol in our hospital: (a) axial 2D T1-weighted imaging (T1WI; TR/TE = 400 ms/14 ms, FOV = 22 × 25 cm, slice thickness = 5 mm, matrix = 192 × 320, number of excitations [NEX] = 1); (b) axial 2D T2WI (TR/TE = 3000/90 ms, FOV = 18 × 20 cm, slice thickness = 3 mm, matrix = 256 × 512, NEX = 2); (c) axial diffusion-weighted imaging (DWI; TR/TE = 5400/73 ms, FOV = 22 × 25 cm, slice thickness = 5 mm, matrix = 128 × 192, b-values = 0, 1000 s/mm^2^, NEX = 1) using single-shot echo planar imaging; (d) three-dimensional (3D) contrast-enhanced T1WI (TR/TE = 5.7/2.21 ms, FOV = 24.2 × 27.5 cm, flip angle = 12°, slice thickness = 1 mm, matrix = 256 × 256); and (e) 3D arterial spin labeling (ASL) imaging (TR/TE = 4897/53.5 ms, FOV = 24 × 27.4 cm, post-label delay = 2000 ms, labeling duration = 1500 ms, slice thickness = 4 mm, number of slices = 40, 512 sampling points on 6 spirals, NEX = 2).

### APT image processing

APT imaging data were analyzed in MATLAB (The MathWorks, Inc., Natick, MA, USA) on an MRI scanner console and the magnetization transfer ratio asymmetry (MTR_asym_) was calculated. Using the shift-corrected data, the MTR_asym_ values at the offset of ± 3.5 ppm with respect to water frequency were calculated as follows [30–32]:

$$MTR_{asym } \left( { + 3.5_{ppm} } \right) = \frac{{S_{sat} \left( { - 3.5_{ppm} } \right) - S_{sat} \left( { + 3.5_{ppm} } \right)}}{{S_{0} }}$$

where S_sat_ is the SI with selective imaging and S0 is the SI in the absence of RF for imaging of SI normalization. B0 field heterogeneity was corrected on a pixel-by-pixel basis using a water-saturation reference map [33].

### Image analysis

APT image date in this study were originally collected before diagnosis and for the purpose of this study it was independently evaluated by two neuroradiologists (with 13 and 37 years of experience in neuroimaging) who were blinded to the clinical data. A circular region of Interest (ROI) was manually placed on a slice of the raw APT imaging that exhibited similar contrast to the T2WI to allow assessment of anatomical landmarks in the brain. The size of was about 5–10 mm, and it was placed in an area that best represented the entire signal of the lesion. Reference was made to both T2WI and post-contrast 3D T1WI to exclude any necrotic portions while including the area with the highest signal within the lesion. The mean MTR_asym_ values of the two readers were used for analysis. Similarly, another ROI was placed in normal-appearing tissue on the opposite side. If lesions are present on the opposite side of the lesion, ROI was placed in normal-appearing white matter.

Conventional MR images were analyzed for tumor location, T1 low intensity, T2 hyperintensity, multiple lesions, contralateral lesions, mass effect, enhancement, poor enhancement area, edema around the lesion, and 3D ASL hyperintensity by the same two neuroradiologists. Tumor locations were categorized as above the tentorium or below the tentorium. T1 low intensity was defined as a lower SI of the tumor relative to gray matter, and T2 hyperintensity was defined as a higher SI of the tumor relative to gray matter. Multiple lesions and contralateral lesions were defined by the presence of multiple distant lesions and the presence of lesions in both hemispheres, respectively, on T2WI and post-contrast 3D T1WI. Mass effect was defined as compression of brain parenchymal structures surrounding the lesion. Enhancement was defined as the presence of enhancement in the lesion, and a poor enhancement area was defined as the presence of a region of poor enhancement within the enhancement area. Therefore, if a lesion showed no enhancement effect, it was also judged as not showing a poor enhancement area in the lesion. Edema around the lesion was defined as the presence of a T2 hyperintense area around the enhanced lesion. Therefore, if the lesion had no enhancement effect, there was also judged to be no edema around the lesion. 3D ASL hyperintensity was defined as higher signal intensity in the lesion relative to surrounding brain parenchyma. If the readers’ diagnoses differed, they reviewed the data to reach a consensus.

### Pathological and reference standard diagnoses

Pathological diagnosis of PCNSL and GBM were made by a neuropathologist at our hospital according to the WHO Histological Classification of Tumours of the Central Nervous System 2021 [34]. PML was diagnosed according to the detection of JCV deoxyribonucleic acid (DNA) in cerebrospinal fluid (CSF) by polymerase chain reaction (PCR).

### Statistical analysis

SPSS for Windows version 24 (SPSS Inc., Chicago, IL, USA) was used to conduct all statistical analyses. The D’Agostino–Pearson test was used to assess the normality of the data, and non-normally distributed variables are presented as the median (range). Quantitative results are expressed as the mean ± SD or median (range).

Age and MTR_asym_ values were analyzed using the Kruskal–Wallis test and Dunn’s test with Bonferroni post hoc correction. Sex, tumor location, multiple lesions, contralateral lesions, mass effect, enhancement, poor enhancement area, edema around the lesion, and 3D ASL hyperintensity were analyzed using the chi-squared test. Results are expressed as sensitivity, specificity, and overall accuracy, with the 95% confidence interval (CI) calculated with the normal approximation method [35].

Receiver operating characteristic (ROC) curves were calculated and used to determine the optimal threshold values for differentiating PML from CNLS and GBM. The intersection of the ROC curve with the bisecting line where sensitivity equaled specificity was considered the optimal threshold. As part of the error analysis, 95% confidence intervals (CI) for each discriminability index will be calculated; 95% CIs for AUC will be calculated using the Wilson method, as it includes results where AUC = 1. The 95% CIs for sensitivity, specificity, positive predictive value, and negative predictive value will be calculated as Clopper–Pearson exact CIs.

Interobserver agreement for MTR_asym_ was evaluated using the intraclass correlation coefficient (ICC), while that for conventional MRI features was evaluated using Cohen's kappa coefficient. ICC and k values > 0.8 and > 0.6 were taken to indicate excellent and good agreement, respectively.

A two-sided *P* value was used for all tests, with *P* < 0.05 being considered statistically significant.

## Results

### Clinical characteristics and imaging findings of PML, PCNSL, and GBM

Of the 22 patients included in this study, 4 patients (18.2%) had PML, 7 patients (31.8%) had PCNSL, and 11 patients (50%) had GBM (Table 1).

**Table 1:** Comparisons of demographics, clinical characteristics, and imaging findings between patients with PML, PCNSL, and GBM

Variable	PML (n = 4)	PCNSL (n = 7)	GBM (n = 11)	*P*
Age (years) ± SD	52.3 ± 6.1	74.4 ± 5.8	65.0 ± 15.2	N.S
Male:female	2:2	3:4	6:5	N.S
Mean MTR_asym_ ± SD (lesion)	0.005 ± 0.005	0.025 ± 0.005	0.025 ± 0.009	0.012
Mean MTR_asym_ ± SD (normal-appearing tissue)	0.003 ± 0.002	0.003 ± 0.002	0.004 ± 0.002	0.389
T1 low intensity	2 (50%)	3 (42.9%)	6 (54.5%)	0.890
T2 hyperintensity	4 (100%)	6 (85.7%)	10 (90.9%)	0.730
Tumor location (above the tentorium)	3 (75%)	6 (85.7%)	11 (100%)	0.279
Multiple lesions	3 (75%)	4 (57.1%)	7 (63.6%)	0.839
Contralateral lesions	3 (75%)	3 (42.9%)	4 (36.4%)	0.408
Mass effect	0 (0%)	7 (100%)	11 (100%)	< 0.001
Enhancement	0 (0%)	7 (100%)	11 (100%)	< 0.001
Poor enhancement area	0 (0%)	0 (0%)	10 (90.9%)	< 0.001
Edema around the lesion	0 (0%)	7 (100%)	10 (90.9%)	< 0.001
3D ASL hyperintensity	0 (0%)	3 (42.9%)	9 (81.8%)	0.014

*SD* standard deviation, *N.S* not significant, *PML* progressive multifocal leukoencephalopathy, *PCNSL* primary central nervous system lymphoma, *GBM* glioblastoma multiforme, *MTR* magnetization transfer ratio, *3D* three-dimensional, *ASL* arterial spin labeling

There was no significant difference in mean MTR_asym_ in normal-appearing tissue (*P* = 0.389), T1 low intensity (*P* = 0.890), T2 hyperintensity (*P* = 0.730), tumor location (*P* = 0.279), multiple lesions (*P* = 0.839), and contralateral lesions (*P* = 0.408) between the three groups.

**Fig. 1:**
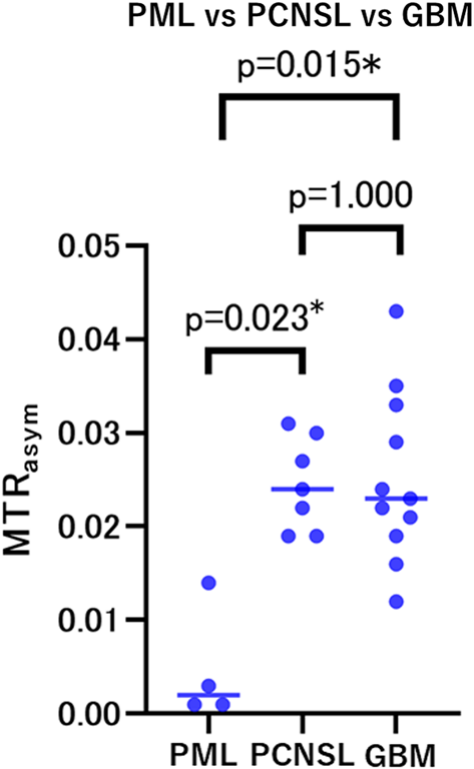
Individual data for MTR_asym_ in patients in the PML, PCNSL, and GBM groups (PML vs PCNSL,* P* = 0.023; PML vs GBM,* P* = 0.015; PCNSL vs GBM,* P* = 1.000). *MTR* magnetization transfer ratio, *PML* progressive multifocal leukoencephalopathy, *PCNSL* primary central nervous system lymphoma, *GBM* glioblastoma multiforme

**Fig. 2:**
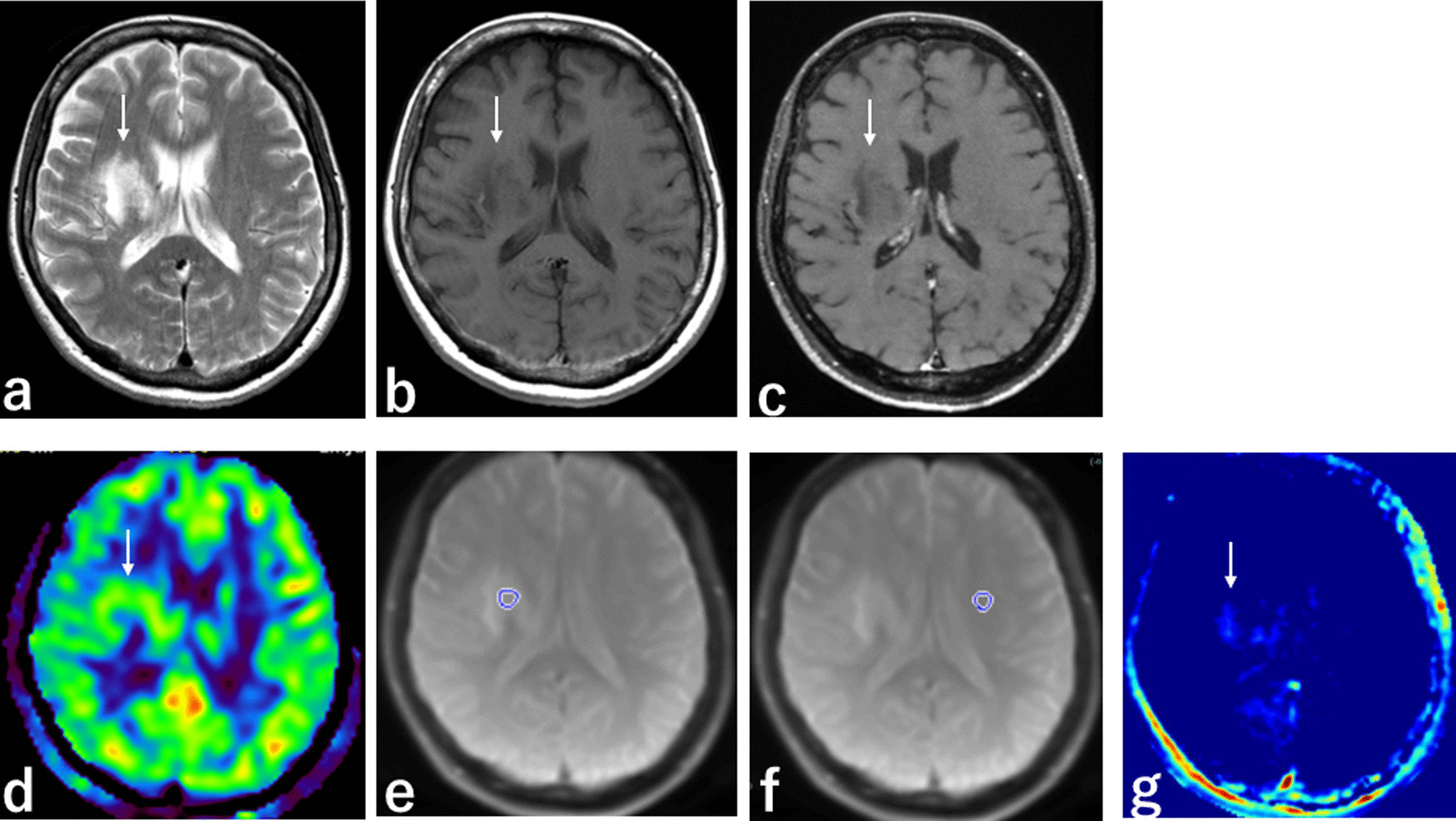
MRI from a 53-year-old woman with PML. **a** T2-weighted sequence, axial section, shows poor definition and high SI in PML in the white matter of the right frontal lobe (white arrow). **b** T1-weighted sequence, axial section, shows poor definition and low SI in PML in the white matter of the right frontal lobe (white arrow). **c** 3D contrast-enhanced T1-weighted sequence, axial section, shows no enhancement in PML. **d** 3D ASL sequence, axial section, shows isointensity in PML. **e** APT scout image, axial section, shows the ROI placed in PML. **f** APT scout image, axial section, shows the ROI placed in normal-appearing tissue contralateral to lesion. **g** APT imaging shows low SI in PML (white arrow). *PML* progressive multifocal leukoencephalopathy, *SI* signal intensity, *ASL* arterial spin labelling, *APT* amide proton transfer

**Fig. 3:**
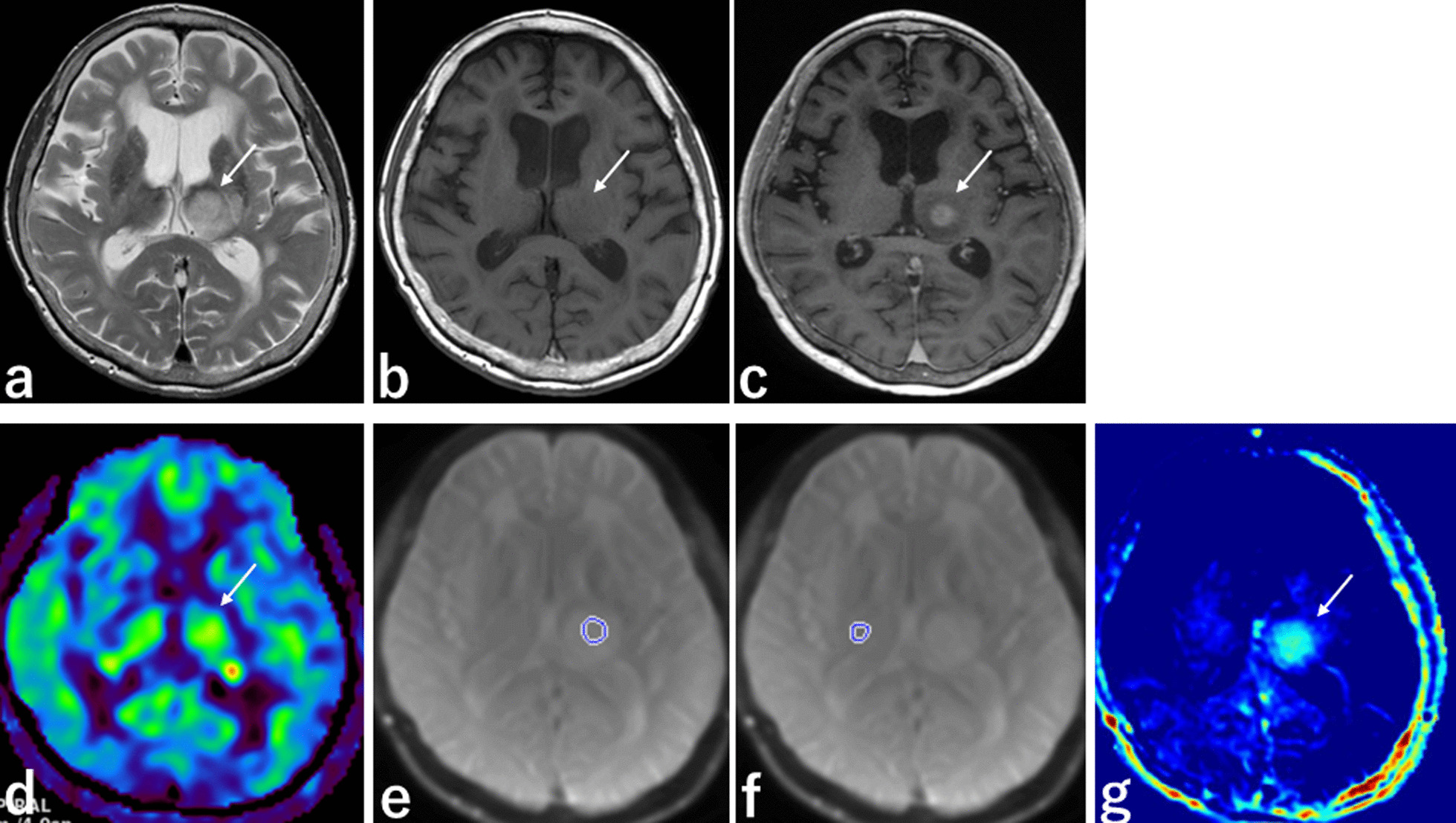
MRI from a 75-year-old woman with PCNSL. **a** T2-weighted sequence, axial section, shows well-defined PCNSL with high SI in the left thalamus (white arrow). **b** T1-weighted sequence, axial section, shows well-defined PCNSL with isointensity in the left thalamus (white arrow). **c** 3D contrast-enhanced T1-weighted sequence, axial section, shows enhancement in the center of PCNSL (white arrows). **d** 3D ASL sequence, axial section, shows isointensity in PCNSL. **e** APT scout image, axial section, shows the ROI placed in PCNSL. **f** APT scout image, axial section, shows the ROI placed in normal-appearing tissue contralateral to lesion. **g** APT imaging shows high SI in PCNSL (white arrow). *PCNSL* primary central nervous system lymphoma, *SI* signal intensity, *ASL* arterial spin labelling, *APT* amide proton transfer

**Fig. 4:**
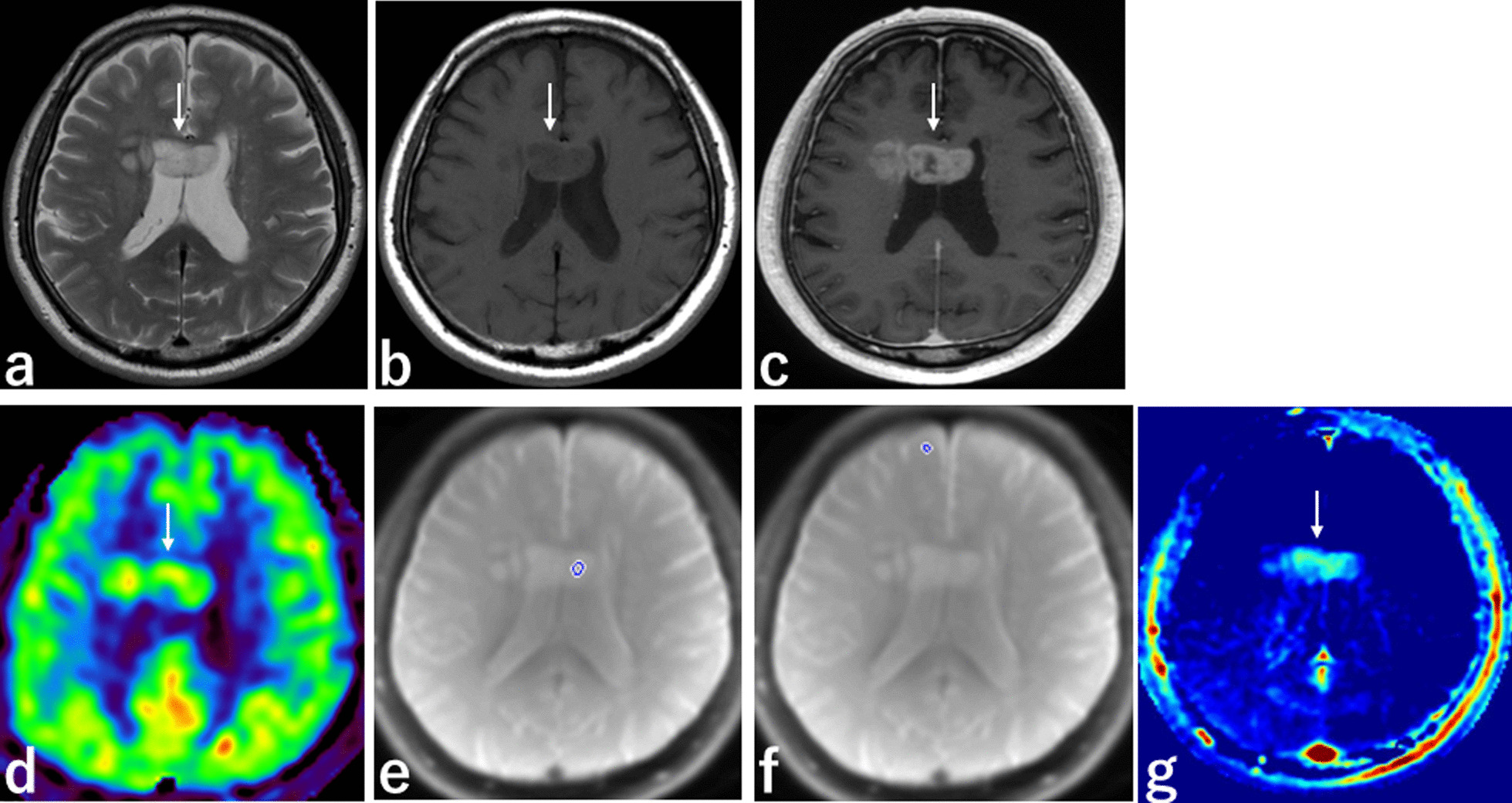
MRI from a 73-year-old man with GBM. **a** T2-weighted sequence, axial section, shows well-defined GBM with high SI in the body of the corpus callosum (white arrow). **b** T1-weighted sequence, axial section, shows well-defined GBM with low SI in the body of the corpus callosum (white arrow). **c** 3D contrast-enhanced T1 sequence, axial section, shows enhancement with a poor enhancement area in GBM (white arrow). **d** 3D ASL sequence, axial section, shows slightly high SI in GBM. **e** APT scout image, axial section, shows the ROI placed in the GBM avoiding the poor enhancement area that may represent a necrotic portion. **f** APT scout image, axial section, shows the ROI placed in normal-appearing tissue in right frontal lobe. **g** APT imaging shows high SI in GBM (white arrow). *GBM* glioblastoma multiforme, *SI* signal intensity, *ASL* arterial spin labelling, *APT* amide proton transfer

Mean MTR_asym_ in the lesion was 0.005 ± 0.005 in the PML group, 0.025 ± 0.005 in the PCNSL group, and 0.025 ± 0.009 in the GBM group, with there being significant differences between the three groups (*P* = 0.012; difference between PML and PCNSL groups, *P* = 0.023; difference between PML and GBM groups, *P* = 0.015). In contrast, there was no significant difference between the PCNSL group and the GBM group (*P* > 0.999) (Fig. 1). Significant differences in mass effect (*P* < 0.001), enhancement (*P* < 0.001), poor enhancement area (*P* < 0.001), edema around the lesion (*P* < 0.001), and 3D ASL hyperintensity (*P* = 0.014) were also found among the three groups. Representative images are shown in Figs. 2, 3 and 4.

### History of the patients with PML

Of the four patients with PML, three had suffered from systemic lupus erythematosus and one from mixed connective tissue disease. All four had taken immunosuppressant drugs before diagnosis with PML.

### Pathological findings

The seven PCNSL cases were diagnosed by brain biopsy performed by neurosurgeons at our hospital, and all were diagnosed with diffuse large B-cell lymphoma. Seven cases of GBM were diagnosed by operation and four were diagnosed by brain biopsy by neurosurgeons at our hospital. Among the eleven cases of GBM, ten were diagnosed as GBM isocitrate dehydrogenase—wild type WHO grade 4, and one was diagnosed as GBM not otherwise specified WHO grade 4.

### ROC analysis for discrimination of PML from PCNSL and GBM

**Fig. 5:**
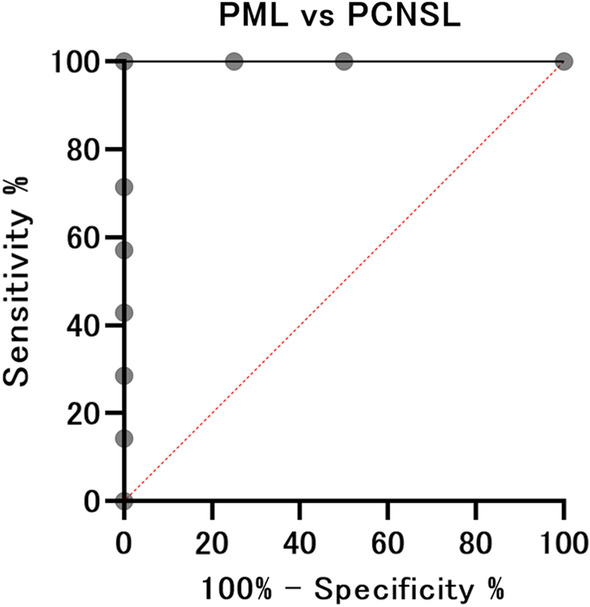
ROC analysis between patients in the PML and PCNSL groups. ROC analyses demonstrated that mean MTR_asym_ has high discriminatory power for differentiating between patients in the PML and PCNSL groups. When an AUC value of < 0.0165 was used as the threshold for diagnosis, the sensitivity, specificity, positive predictive value, and negative predictive value were all 100%. *ROC* receiver operating characteristics, *PML* progressive multifocal leukoencephalopathy, *PCNSL* primary central nervous system lymphoma, *MTR* magnetization transfer ratio, *AUC* area under the curve

**Fig. 6:**
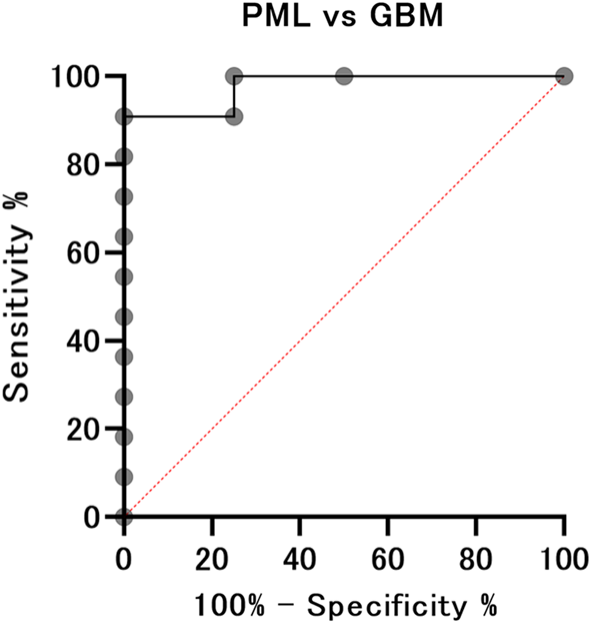
ROC analysis between patients in the PML and GBM groups. ROC analyses demonstrated mean MTR_asym_ to have high discriminatory power for differentiating between patients in the PML and GBM groups. When an AUC value of < 0.015 was used as the threshold for diagnosis, the sensitivity, specificity, positive predictive value, and negative predictive value were 90.9%, 100%, 100%, and 96.8%, respectively. *ROC* receiver operating characteristics, *PML* progressive multifocal leukoencephalopathy, *GBM* glioblastoma multiforme, *MTR* magnetization transfer ratio, *AUC* area under the curve

ROC analyses demonstrated that mean MTR_asym_ had high discriminatory power (area under the curve [AUC] = 1.00 [95% CI 0.720–1.000]) for differentiating patients with PML from those with PCNSL. When a cut-off value of < 0.0165 was used as the threshold for diagnosis, the sensitivity, specificity, positive predictive value, and negative predictive value were all 100% (95% CI 39.8–100%, 59.0–100%, 39.8–100%, and 59.0–100%, respectively) (Fig. 5). ROC analyses also demonstrated that mean MTR_asym_ had high discriminatory power (AUC = 0.98 [95% CI 0.852–1.000]) for differentiating patients with PML from those with GBM. When a cut-off value < 0.015 was used as the threshold for diagnosis, the sensitivity, specificity, positive predictive value, and negative predictive value were 100%, 90.9%, 80.0%, and 100% (95% CI 39.8–100%, 58.7–99.8%, 28.4–99.5%, and 69.2–100%), respectively (Fig. 6).

### Interobserver agreement

Interobserver agreement for measurement of MTR_asym_ was excellent, with ICC values of 0.990 [95% CI, 0.982–0.995]. Interobserver agreement for specific conventional imaging features was good to excellent for all parameters (k values: 0.704–1.000).

## Discussion

When patients are in an immunocompromised condition, reactivation of JCV can occur, leading to PML. While PML was originally observed in patients with advanced human immunodeficiency virus (HIV) infection, a new category of PML has recently emerged among patients treated with immunomodulatory medications for autoimmune diseases [36–40].

The diagnosis of PML requires detection of JCV DNA in the CSF by PCR, and occasionally brain biopsy. However, MRI is the first modality of choice for diagnosing PML. The MRI features described as representative of PML include hyperintense lesions on T2WI and FLAIR imaging that are asymmetric and are without edema, mass effect, and contrast enhancement [5]. PCNSL and GBM usually show edema around the lesion, a mass effect, and/or contrast enhancement [41–44], and these MRI findings are therefore useful for differentiating PCNSL and GBM from PML. In this study, there were significant differences in mass effect (*P* < 0.001), enhancement (*P* < 0.001), edema around the lesion (*P* < 0.001), and 3D ASL hyperintensity (*P* = 0.014) among the three groups. However, contrast enhancement on MRI can be seen in some cases of PML associated with HIV [45]. Furthermore, MRI contrast agents cannot be used for all patients because of renal dysfunction and other contraindications.

Several studies showed the utility of APT imaging for evaluating brain tumors [24, 28, 46], and as APT imaging does not require contrast agent, it is safe for use in patients with renal failure or those who show adverse reactions to contrast media. However, to the best of our knowledge, our study is the first to evaluate APT imaging for differentiating PML from PCNSL and GBM.

Previous studies on gliomas demonstrated a positive correlation of APT SI with cell proliferation index [21, 23]. This suggest that high-grade tumors, which show high proliferation, have higher cellular protein and peptide contents than low-grade tumors, and that the cellular concentration of mobile proteins and peptides may increase with the grade of glioma. Other possible contaminations are such as T1 and nuclear Overhauser enhancement. But the contamination from T1 should be small compared with APT with the high saturation (use the micro symbol in 2uT) and relatively shorter saturation time (2 s) as shown previously [47, 48].

We are not aware of any reports on APT imaging of PML. PML is induced by JCV infection and replication, which leads to necrosis of oligodendrocytes. Pathologically, PML is characterized by widespread multifocal demyelination. Therefore, this lesion is not a tumor, and we expected that the APT SI would be low, because the proliferation ability of PML lesions is probably low.

In this study, the mean MTR_asym_ in the PML group was significantly lower than in the PCNSL and GBM groups, and ROC analyses demonstrated that mean MTR_asym_ had high discriminatory power for differentiating patients with PML from those with PCNSL or GBM. However, there was no significant difference in mean MTR_asym_ between the PCNSL and GBM groups. A previous report found that PCNSL showed more homogeneous APT SI than high-grade glioma, and that the maximum APT SI in PCNSL was lower than in high-grade glioma [49]. In this study, MTR_asym_ was more variable in the GBM group than in the PCNSL group, and the MTR_asym_ of some cases in the GBM group was higher than that of the PCNSL group. The lower maximum APT SI values in the PCNSL group compared with the GBM group may reflect the histological characteristics of higher nucleo/cytoplasmic ratios, with less cytoplasm volume and more nuclei in PCNSLs than in GBM [50–52]. Moreover, unlike GBM, PCNSLs are histologically relatively homogenous [53, 54], which may be reflected by the APT SI homogeneity in the PCNSL group. On the other hand, APT SI values in GBM in this study are lower than reported in the literature [21]. A possible reason is that the signal in GBM is heterogeneous and higher APT signal was present in other cross-sections, which may have been outside the ROI in this study.

In this study, PML tended to show lower APT SI than PCNSL or GBM. Our results suggest that APT SI may enable PML to be distinguished from PCNSL and GBM.

This study has several limitations. First, our study included a small number of patients with PML, PCNSL, and GBM, and lacked an external validation cohort. However, the results clearly demonstrate the potential of APT imaging, and we present this study as a pilot study with a plan for a larger follow-up one. Second, the PML sizes are small and scattered, so it is possible that the MTR_asym_ is lower due to the partial volume effect. Third, instead of evaluating entire lesions, ROIs were placed manually within the lesions where we thought the ROI best represented the lesion. We chose this method using the APT images because some lesions occurred near the skull, which is susceptible to field inhomogeneity that may affect the APT SI. We also used an SSFSE sequence for the APT imaging, to reduce field inhomogeneity problems. A last point is that the resolution of APT imaging must be improved to achieve adequate coregistration with conventional MRI images.

## Conclusions

PML exhibited significantly lower APT SI than PCNSL or GBM. Therefore, APT imaging can provide additional quantitative information for the diagnosis of PML, and may help differentiate it from PCNSL and GBM. However, our results need to be validated in a larger follow-up study.

## Data Availability

The datasets during this study are not publicly available, but are available from the corresponding author on reasonable request.

## Abbreviations

2D: Two-dimensional
3D: Three-dimensional
AIDS: Acquired immunodeficiency syndrome
ASL: Arterial spin labelling
APT: Amide proton transfer
AUC: Area under the curve;
CEST: Chemical exchange saturation transfer
CI: Confidence interval
DLBLC: Diffuse large B-cell lymphoma
DNA: Deoxyribonucleic acid
DWI: Diffusion-weighted imaging
EPI: Echo planar imaging
FLAIR: Fluid attenuated inversion recovery
FOV: Field of view
GBM: Glioblastoma multiforme
ICC: Intraclass correlation coefficient
JCV: John Cunningham virus
MRI: Magnetic resonance imaging
MTR: Magnetization transfer ratio
NEX: Number of excitations
PCNSL: Primary central nervous system lymphoma
PML: Progressive multifocal leukoencephalopathy
RF: Radiofrequency
ROC: Receiver operating characteristics
ROI: Region of interest
SD: Standard deviation
SI: Signal intensity
Ssat: Signal intensity with selective imaging
SSFPE: Single-shot fast spin echo
T1WI: T1-weighted imaging
T2WI: T2-weighted imaging
TE: Echo time
TR: Repetition time
WHO: World Health Organization
